# Joint modelling of longitudinal and time-to-event data: an illustration using CD4 count and mortality in a cohort of patients initiated on antiretroviral therapy

**DOI:** 10.1186/s12879-020-04962-3

**Published:** 2020-03-30

**Authors:** Nobuhle N. Mchunu, Henry G. Mwambi, Tarylee Reddy, Nonhlanhla Yende-Zuma, Kogieleum Naidoo

**Affiliations:** 1 Biostatistics Unit, South African Medical Research Council (SAMRC), SAMRC Building, 491 Peter Mokaba Ridge Road, Durban, 4041, South Africa; 2 University of KwaZulu-Natal, School of Mathematics, Statistics and Computer Science, King Edward Avenue, Pietermaritzburg, 3209, South Africa; 3 Centre for the AIDS Programme of Research in South Africa (CAPRISA), University of KwaZulu-Natal, 719 Umbilo Road, Durban, 4041, South Africa; 4 MRC-CAPRISA HIV-TB Pathogenesis and Treatment Research Unit, Doris Duke Medical Research Institute, University of KwaZulu-Natal, Durban, South Africa

**Keywords:** Time-to-event data, Longitudinal data, Joint models, CD4 count, Mortality, Bias

## Abstract

**Background:**

Modelling of longitudinal biomarkers and time-to-event data are important to monitor disease progression. However, these two variables are traditionally analyzed separately or time-varying Cox models are used. The former strategy fails to recognize the shared random-effects from the two processes while the latter assumes that longitudinal biomarkers are exogenous covariates, resulting in inefficient or biased estimates for the time-to-event model. Therefore, we used joint modelling for longitudinal and time-to-event data to assess the effect of longitudinal CD4 count on mortality.

**Methods:**

We studied 4014 patients from the Centre for the AIDS Programme of Research in South Africa (CAPRISA) who initiated ART between June 2004 and August 2013. We used proportional hazards regression model to assess the effect of baseline characteristics (excluding CD4 count) on mortality, and linear mixed effect models to evaluate the effect of baseline characteristics on the CD4 count evolution over time. Thereafter, the two analytical approaches were amalgamated to form an advanced joint model for studying the effect of longitudinal CD4 count on mortality. To illustrate the virtues of the joint model, the results from the joint model were compared to those from the time-varying Cox model.

**Results:**

Using joint modelling, we found that lower CD4 count over time was associated with a 1.3-fold increase in the risk of death, (HR: 1.34, 95% CI: 1.27-1.42). Whereas, results from the time-varying Cox model showed lower CD4 count over time was associated with a 1.2-fold increase in the risk of death, (HR: 1.17, 95% CI: 1.12-1.23).

**Conclusions:**

Joint modelling enabled the assessment of the effect of longitudinal CD4 count on mortality while correcting for shared random effects between longitudinal and time-to-event models. In the era of universal test and treat, the evaluation of CD4 count is still crucial for guiding the initiation and discontinuation of opportunistic infections prophylaxis and assessment of late presenting patients. CD4 count can also be used when immunological failure is suspected as we have shown that it is associated with mortality.

## Background

Until the era of test-and-treat, CD4 count was by far the most widely used biological marker for antiretroviral therapy (ART) eligibility and HIV (human immunodeficiency virus) progression [1]. However, the introduction of universal test and treat has put less emphasis on the importance of CD4 count and only viral load is now used to monitor HIV disease progression and virologic failure. Arguably, the role of CD4 count in the current era of HIV monitoring is still crucial, particularly for patients presenting late to care as they are at high risk of presenting with opportunistic infections and also in areas where viral load testing is not affordable [2, 3].

Although CD4 count testing is no longer recommended for stable virologically suppressed patients in South Africa, however, the CD4 count still plays an important role in stratifying the risk of death among patients with low CD4 count who are failing first line ART regimen [4]. In South Africa, it was estimated that 7.9 million people of all ages were living with HIV in 2017 [5]. The country has the largest ART programme in the world with 4.7 million on lifelong ART treatment and these efforts have been largely financed from its own domestic resources [6]. Considering that South Africa is not a rich resourced country, managing such high volume of ART patients requires resources and different biomedical strategies using different longitudinal biomarkers at different stages of the disease.

In our setting, it has been shown that patients who started ART with low CD4 count have an increased risk of death [7] and it is in keeping with research from other settings [8]. Even though longitudinal CD4 count was not used in these findings, one can deduce that these patients’ CD4 count recovery over time would have been slow and hence they died. Statistical models of the association between longitudinal CD4 count and mortality have been carried out using time-varying Cox models [9]. The counting process formulation of the time-varying Cox model has a distinct flexibility that not only allows for time-dependent covariates but also for left truncation, multiple time scales, multiple events per subject and various forms of case-cohort models, among others [10]. However, this model assumes that time-dependent covariates are (i) measured without error and (ii) are external or exogenous (that is, the value of the covariate at a future time point is not affected by the occurrence of the event). The second assumption is not valid for endogenous covariates (such as clinical biomarkers) [11], since the repeatedly measured marker like CD4 count is directly related to the mortality mechanism. Longitudinal and time-to-event outcomes are traditionally analyzed separately, however this approach leads to inefficient or biased results for the time-to-event model [12], due the fact that this type of modelling fails to recognize the shared random-effects from the two processes. The longitudinal model accounts for measurement error by postulating that the observed level of the longitudinal outcome equals the true level plus a random error term. Thus joint models are preferred over separate analyses and time-dependent models because they account for the special features of endogenous covariates and non-random dropout in a longitudinal data analysis context [11, 13].

Joint models of longitudinal and time-to-event data have received much attention in the literature dating back to the past two decades [14–16], and have been considered within the HIV context [17–21], however its application is limited when data from the sub-Saharan African region is used for modelling.

In view of the shortcomings of time-varying Cox models and separate analyses of longitudinal and time-to-event processes, our objective is to use a joint modelling strategy to assess the effect of longitudinal CD4 count on mortality among patients initiated on ART.

## Methods

### Source of data and description

In this analyses we use data from the Centre for the AIDS Programme of Research in South Africa (CAPRISA). The CAPRISA AIDS Treatment (CAT) programme enrolled HIV positive patients and initiated them on ART between June 2004 and August 2013. Eligibility criteria was in accordance with the Department of Health guidelines throughout. Males and females at least 14 years of age from urban (eThekwini) and rural (Vulindlela) sites were enrolled. Routine demographic and clinical data were recorded at baseline and at follow-up visits. Laboratory safety assessments and CD4 counts and viral loads were conducted at baseline and every 6 months or as clinically indicated. Patients were regarded as lost to follow-up if they missed 3 consecutive scheduled visits and if all attempts to track them telephonically and physically had failed. Information on the deaths was based on hospital chart notes, death certificates or oral reports from patient’s relatives.

The eThekwini and Vulindlela sites initiated the first patient on ART in October 2004 and June 2004 respectively. Patients at the eThekwini site were recruited from the Prince Cyril Zulu Clinic of Communicable Disease which is the chest clinic adjacent to the CAPRISA clinic and sometimes patients presented themselves for HIV testing. Patients at the Vulindlela site were recruited from the Mafakatini clinic which is situated near that site or similarly presented themselves seeking health care.

### Statistical analysis

In our setting it has been shown that gender is associated with both CD4 count and mortality [7]. Therefore, descriptive data, which was stratified by gender were presented as medians with interquartile range, percentages and graphical exploration was used where applicable. Unpaired t-test or the Wilcoxon rank sum test was used to compare continuous demographics and clinical data for men and women. Fisher’s exact test was used for the comparison of categorical data. Poisson regression was used to calculate 95% confidence intervals (CI) for mortality rates and F-test was used for their comparisons.

Baseline predictors of mortality were assessed through both univariable and multivariable proportional hazards regression. The linear mixed effects models were used to assess the effect of baseline characteristics on the CD4 count evolution post ART initiation, where the individual patient and time post ART initiation were used as random effects. All multivariable models were adjusted for the following baseline covariates: gender (male or female), age (in years), clinic site (urban and rural), log_10_ viral load and tuberculosis (TB) status. A square root transformation was applied to the data to normalize the CD4 count.

Proportionality was assessed by the Schoenfeld proportional hazards test which provides proportional hazards test for individual covariates and a global test for the model with all variables combined. Variables that violated the proportional hazards assumption were not included in the proportional hazards model.

Thereafter the longitudinal and time-to-event models were coupled to form a joint model. To illustrate the virtues of the joint model we compared it to the time-varying Cox model. Analyses were conducted using SAS, version 9.4 (SAS Institute INC., Cary) and R version 3.5.1. The JM package by [11] was used to fit the joint model.

### The joint model formulation

Formulating a standard joint modelling framework, follows a typical setup where you have a linear mixed-effects (LME) model for the longitudinal data and a Cox proportional hazards (PH) model for the time-to-event data, with the two models sharing some random effects [22]. This is the so called shared parameter model approach.

#### The longitudinal sub-model

Assume *b*_*i*_∼*N*(***0***,***Σ***_*b*_) then

1 $$ y_{ij}=\mathbf{X}_{i}[t_{ij}]^{'}\boldsymbol{\beta}+b_{0i}+b_{1i}t_{ij}+\epsilon_{ij}  $$

with mutually independent error $\epsilon _{ij} \sim N(0,\sigma ^{2}_{\epsilon })$. Furthermore *ε*_*ij*_ is taken as independent of the random intercept *b*_0*i*_ and slope *b*_1*i*_.

#### The survival sub-model

The Cox proportional hazards model with a covariate described by the random effects model given above is written as

2 $$ \lambda(t|\boldsymbol{b}_{i},\boldsymbol{y}_{i})\,=\,\lambda(t|\boldsymbol{b}_{i})\,=\,\lambda_{0}(t)\exp\{(\mathbf{X}_{i}[t]^{'}\boldsymbol{\boldmath\beta}+b_{0i}+b_{1i}t)\gamma\}.  $$

Estimates for this semi-parametric approach are obtained by the Expectation-maximization (EM) algorithm [15].

#### The joint likelihood

Assuming non-informative censoring and a measurement schedule *t*_*ij*_, that is independent of the random effects and covariate history, the joint likelihood $L(\boldsymbol {\theta })=L(\boldsymbol {\beta },\boldsymbol {b},\boldsymbol {\Sigma }_{b},\sigma ^{2}_{\epsilon },\lambda _{0})$ for event time data and longitudinal measurements is given by

3 $$ \begin{aligned} L(\boldsymbol{\theta}) & =\boldsymbol{\prod_{i=1}^{k}} \bigg[\int \biggl\{\boldsymbol{\prod_{j=1}^{n_{i}}}f(y_{ij}|\boldsymbol{b}_{i},\boldsymbol{t}_{i},\sigma^{2}_{\epsilon})\biggr\} \\ & \times f(\tau^{*}_{i},\delta_{i}|\boldsymbol{b}_{i},\boldsymbol{t}_{i},\lambda_{0},\boldsymbol{\beta})f(\boldsymbol{b}_{i}|\boldsymbol{\Sigma}_{b})d\boldsymbol{b}_{i}\bigg] \end{aligned}  $$

where $f(y_{ij}|\boldsymbol {b}_{i},\boldsymbol {t}_{i},\sigma ^{2}_{\epsilon })$ and *f*(***b***_*i*_|***Σ***_*b*_) are the densities of *y*_*ij*_ and *N*(***0***,***Σ***_*b*_) respectively and

4 $$ \begin{aligned} f(\tau^{*}_{i},\delta_{i}|\boldsymbol{b}_{i},\boldsymbol{t}_{i},\lambda_{0},\beta) & = \{\lambda_{0}(\tilde{\tau}^{*}_{i}(\beta,y_{i}))\exp[y_{i}(\tau^{*}_{i})\beta]\}^{\delta_{i}} \\ & \times \exp\biggl\{-\int_{0}^{\tilde{\tau}^{*}_{i}(\beta,y_{i})}\lambda_{0}(t)dt\biggr\} \end{aligned}  $$

with $\tilde {\tau }^{*}_{i}(\beta,y_{i})=\int _{0}^{\tau ^{*}_{i}}\exp [y_{i}(s)\beta ]ds.$

## Results

### Exploratory data analysis at ART initiation

**Fig. 1:**
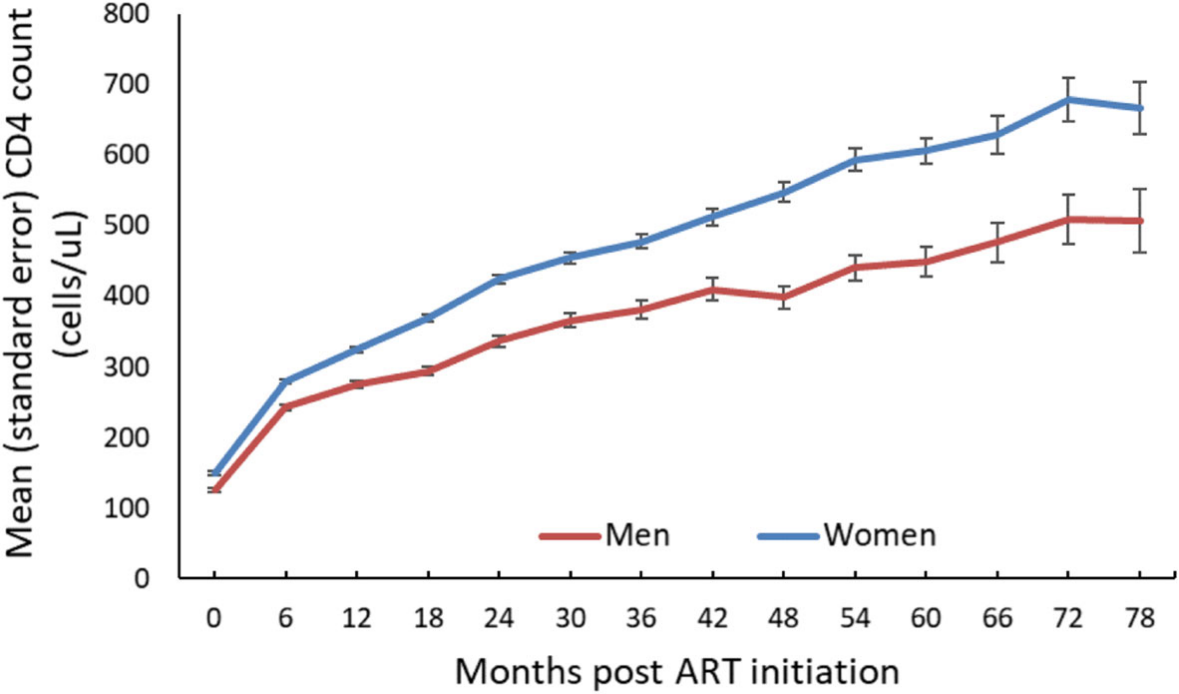
Mean CD4 count (cells/ *μ*L) over time by gender

**Fig. 2:**
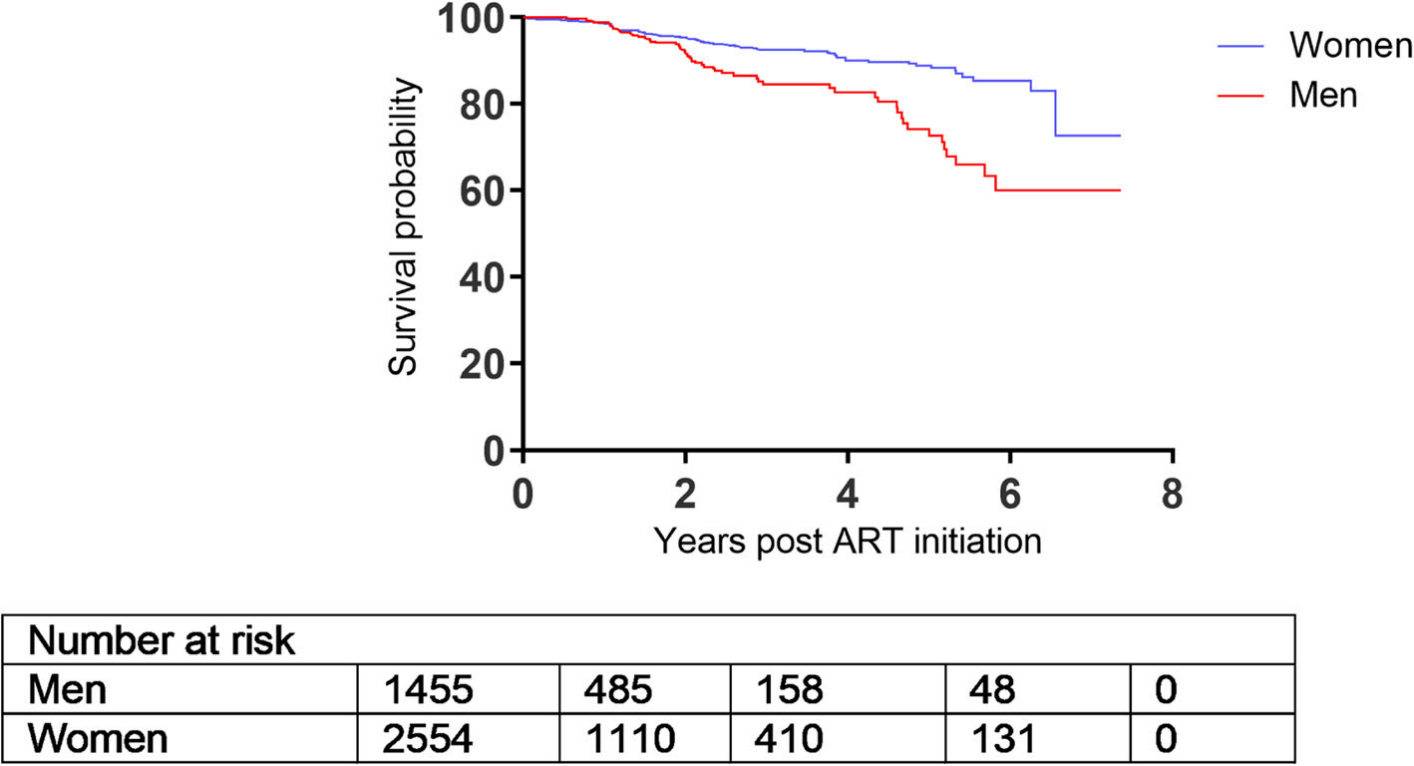
Kaplan-Meier curve for survival by gender

**Fig. 3:**
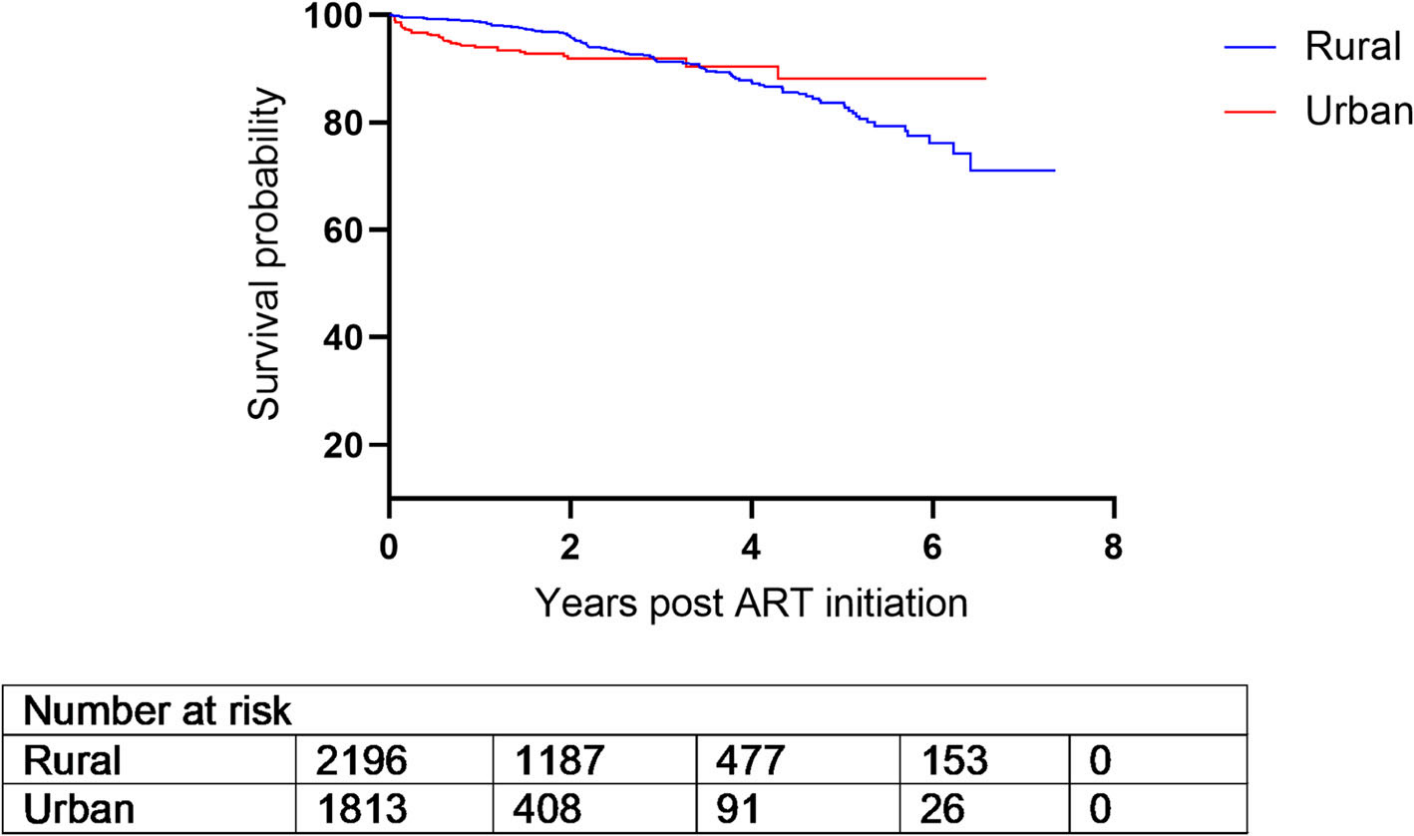
Kaplan-Meier curve for survival by site

**Fig. 4:**
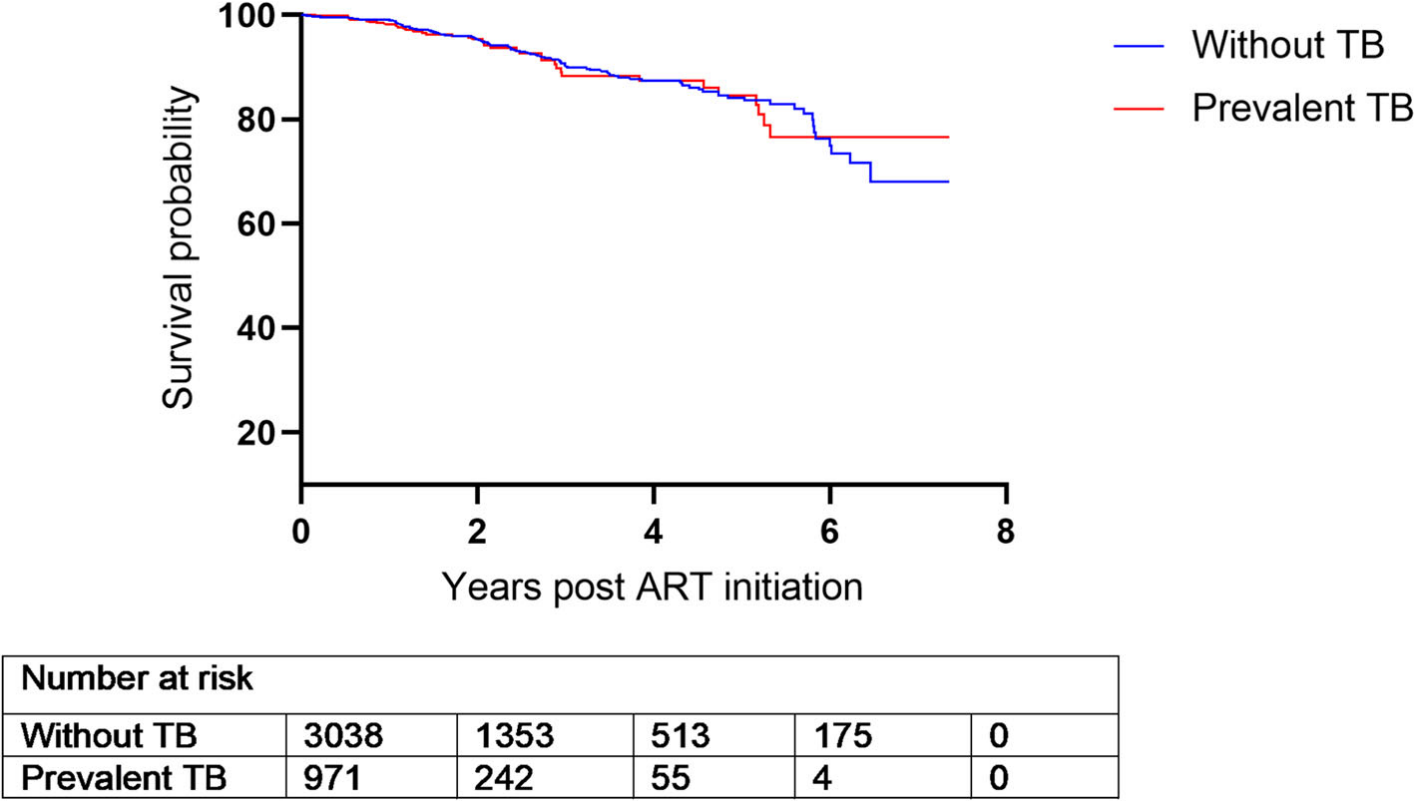
Kaplan-Meier curve for survival by TB status

**Fig. 5:**
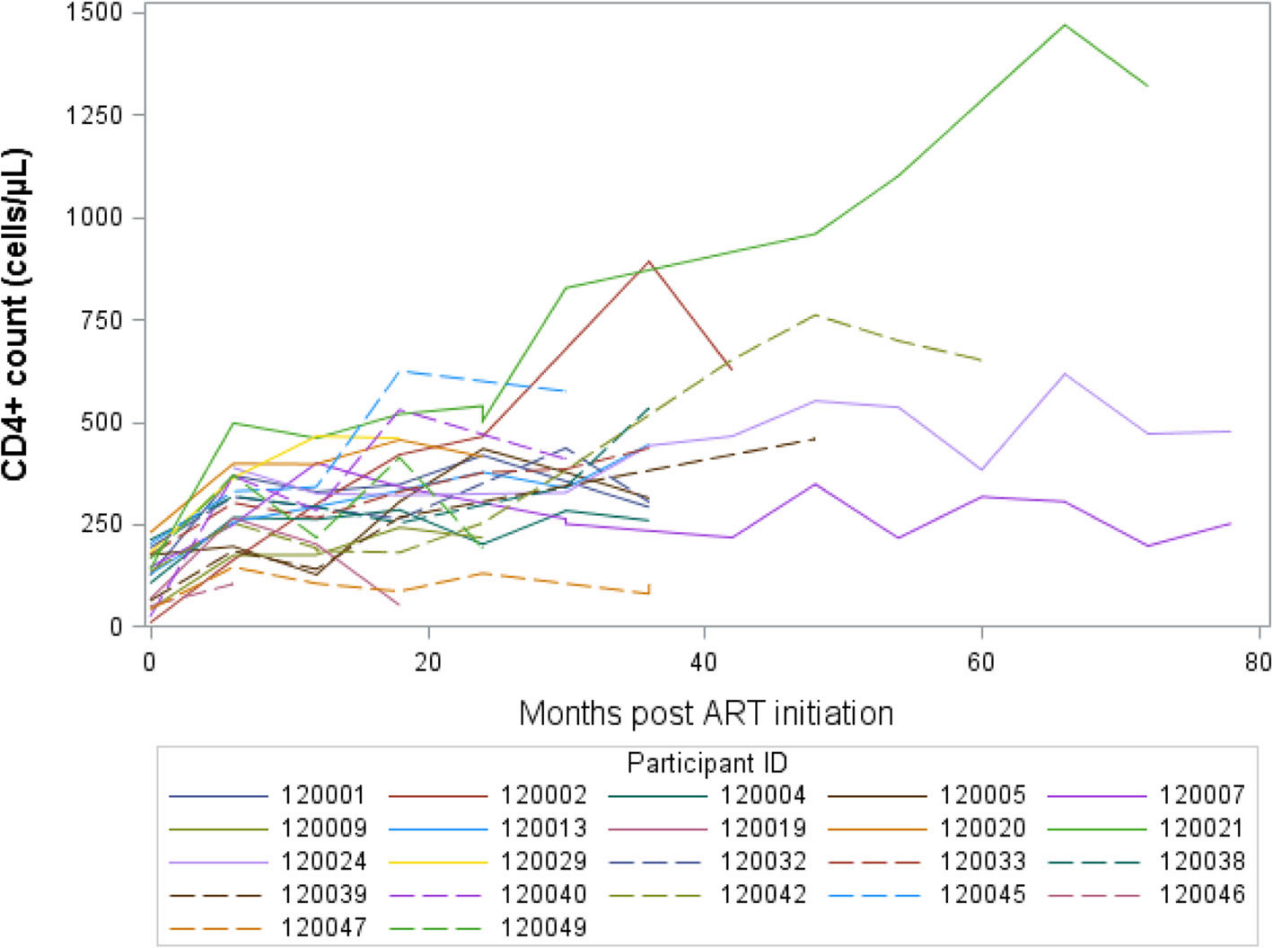
CD4 count trajectories over time

There were 4014 patients enrolled whose ages range from 14–76 (with an overall mean age of 34.6 years). Out of the 4014, 2557 (63.7%) were females. TB prevalence was higher in men compared to women (32.1% vs. 19.7%). Moreover, women initiated ART with slightly higher CD4 count than men (132.0 vs. 113.0 cells/mm^3^, p <0.001) (Table 1), and this pattern persisted over time (Fig. 1) and matches the probability of death (Fig. 2). Patients from the urban site have a lower probability of death when compared to those from the rural site (Fig. 3) in addition, patients presenting without TB at ART initiation have a lower survival prognosis compared to those with prevalent TB (Fig. 4). Figure 5 depicts an increasing trend of CD4 count over time after ART initiation for 22 randomly selected patients. What can be observed is that generally there is evidence of between subjects variability as well as within subject variability. The subjects have large CD4 count evolutions over time, this suggests that perhaps linear mixed models with random intercepts and slopes could be plausible starting points.

**Table 1:** Baseline characteristics of patients initiated on ART

Characteristic	Women (N= 2557)	Men (N= 1457)	*p*-value
Age (years), median (IQR) ^*a*^	32.0 (28.0-39.0)	35.0 (30.0-41.0)	<0.001
Site, n (*%*):			<0.001
Rural	1507 (58.9)	692 (47.5)	
Urban	1050 (41.1)	765 (52.5)	
Prevalent TB?, n (*%*):			<0.001
No	2052 (80.3)	990 (67.9)	
Yes	505 (19.7)	467 (32.1)	
Body mass index (kg/m^2^),			
median (IQR) ^*b*^	24.2 (21.0-28.1)	21.0 (19.0-23.2)	0.415
CD4 count (cells/ *μ*L),			
median (IQR) ^*c*^	132.0 (69.0-202.0)	113.0 (47.0-177.0)	<0.001
CD8 count (cells/ *μ*L),			
median (IQR) ^*d*^	818.5 (533.5-1197.5)	736.0 (462.0-1123.0)	<0.001
Viral load (log_10_ copies/ml),			
mean (SD) ^*e*^:	4.9(0.9)	5.0 (0.9)	<0.001
CD4:CD8 ratio, median(IQR) ^*f*^	0.2 (0.1-0.2)	0.1 (0.1-0.2)	<0.001

^*a*^4 patients had missing age, ^*b*^237 patients had missing BMI, ^*c*^382 patients had missing CD4 count, ^*d*^1936 patients had missing baseline CD8 count, ^*e*^488 patients had missing baseline viral load, ^*f*^1929 patients had missing CD4:CD8 ratio

There were 161 (11,1%) and 235 (9.2%) men and women lost to follow-up respectively, with men having higher lost to follow-up rates in our cohort in keeping with reports from published literature [23–25]. Moreover, patients who were lost to follow-up had a mean of 33.0 years of age and had on average, a CD4 count of 135.8 cells/ *μL* compared to a mean CD4 count of 140.7 cells/ *μL* for patients who were not lost to follow-up. In addition, patients were lost to follow up at a median (IQR) of 1.7 (0.7-3.1) years, men and women were lost to follow-up at a median (IQR) of 1.5 (0.5–2.9) and 1.9 (0.9–3.2) years respectively (p = 0.118).

Out of the 4014 patients, 190 had only one CD4 measurement and the baseline mean CD4 count among these patients was 196.3 compared to a baseline mean CD4 count of 137.1 among patients with more than one CD4 count measurement. Of the 190 patients with only one CD4 count measurement, 34 (17.9%) died soon after this measurement was taken at a median time to death of 0.66 years. Among patients with only one CD4 count measurement, 27 (14,2%) presented with WHO clinical stage 1, 56 (29.5) presented with WHO clinical stage 2, 89 (46.8%) presented with WHO clinical stage 3 and 18 (9.5%) presented with WHO clinical stage 4. Among patients with more than one CD4 count measurement, 552/3799 (14.5%) presented with WHO clinical stage 1, 750/3799 (19.7%) presented with WHO clinical stage 2, 2057/3799 (54.15%) presented with WHO clinical stage 3 and 440/3799 (11.6%) presented with WHO clinical stage 4.

### Results from the longitudinal sub-model: random effects multivariable model

Men started ART with low CD4 count (*β*= -1.90, S.E= 0.22, p <0.001) when compared to women. However, their CD4 count evolution was not significantly different and thus the interaction term between gender and time was excluded in the model (Table S1). Patients presenting without TB at ART initiation started ART with higher mean CD4 count compared to those with prevalent TB but their rate of increase in CD4 count was slower when compared to those with prevalent TB (*β*= -1.27, S.E= 0.17, p <0.001).

### Mortality rates

There were a total of 414 deaths observed over 8195.87 person-years of follow-up. Mortality rates for men and women were 6.7 (95% CI: 5.8- 7.8) and 4.3 (95% CI: 3.8- 4.9) per 100 person-years (p-y), respectively; mortality rate ratio (MRR): 1.54 (1.27-1.88) p <0.001. In addition, patients from the rural site had higher mortality rates compared to those from the urban site, 4.8 per 100 p-y, (95% CI: 4.3-5.4) vs. 5.6 per 100 p-y, (95% CI: 4.7-6.6); MRR: 1.16, (95% CI: 0.95-1.42), *p*= 0.156.

### Results from the survival sub-model: modelling mortality

The multivariable proportional hazards regression analysis showed that men had a 62% significantly elevated risk of death when compared to women, HR: 1.62, (95% CI: 1.16-2.26), *p*= 0.005. In addition, patients who started ART with a higher baseline viral load had a significantly higher risk of death HR: 1.57, (95% CI: 1.26-1.96), *p*= 0.004 (Table S2).

### Results from the joint model of longitudinal and time-to-event data

The joint model finds a significantly strong association between the CD4 count and the risk of death, with a unit decrease in the square root CD4 count corresponding to a 1.3-fold increase in the risk of death (HR: 1.34, 95% CI: 1.27-1.42). These results are statistically significant indicating that indeed CD4 count is a good predictor of mortality and in fact confirms that an increase in CD4 counts is associated with better survival (Table 3). These results were compared with those from the time-varying Cox model and we also observe a strong association between the longitudinal CD4 count and the risk of death. In particular a unit decrease in the square root CD4 count corresponds to a 1.2-fold increase in the risk of death (HR: 1.17, 95% CI: 1.12-1.23) (Table 3). Previous research and simulation studies have shown that the time-varying Cox model underestimates the true association size of markers [11].

## Discussion

Results from the longitudinal sub-model (random effects multivariable model), showed no statistical difference between the urban and rural sites in terms of the CD4 count improvement over time, with patients from the urban site having a higher rate of change. This finding reaffirms the results obtained by [7, 26, 27]. Men and older people on average initiated ART with significantly lower CD4 counts. These results support those obtained by [28, 29]. Patients presenting without TB at ART initiation started ART with high mean CD4 count compared to those with prevalent TB but their rate of change in CD4 count was significantly less compared to those with prevalent TB. These results are similar to those found by [7] in the same study.

Results from the survival sub-model (Cox proportional hazards regression analysis) showed that patients from the urban site had a higher survival prognosis compared to those from the rural site, however this was not significant at 5% level of significance. Patients presenting without TB at ART initiation had an elevated risk of dying compared to those with prevalent TB. These results reaffirms the results obtained by [26]. Prevalent TB was also previously shown to be associated with low mortality, maybe related to TB care being an access point to earlier ART initiation [7, 26]. Published literature has cited that undiagnosed TB is higher among patients accessing ART than in the general population; with the majority of incident TB diagnosed in the early weeks of ART initiation being TB prevalent but missed at baseline screening [30]. In addition male patients, older people or those with a higher mean baseline viral load had a significantly elevated risk of death (refer to Table S2). This finding is in consonance with previous research which showed that men and older patients were at an increased risk of mortality due to HIV/AIDS [26, 28, 31].

The joint model was advantageous for answering multivariate questions at the same time (in our case CD4 count and mortality). The most appealing feature of joint models is its ability to capture or take into consideration the association between the survival time and repeated measurement of a risk factor variable [11]. The joint model showed a significantly strong association between CD4 count and the risk for death, implying that CD4 count is a good predictor of mortality. The joint model also helped assess the correlation between the two response variables and gave ample opportunity to see predictors of the two response variables simultaneously. The results in this study indicated that CD4 count change due to ART and mortality had been influenced jointly by some of the covariates like gender, age, baseline viral load, time (in years) and by the interaction effects of time (in years) with TB status and baseline viral load (refer to Tables 2 and 3). Research findings from a longitudinal study by [13] also showed that CD4 count change was affected by these covariates.

**Table 2:** Longitudinal process estimates from the joint model

	*β* estimate ^*a*^	S.E.	*p*-value
Intercept	18.26	0.73	<0.001
Age (years)	-0.02	0.01	0.052
Men (ref: women)	-1.86	0.22	<0.001
Urban site (ref: rural)	-0.27	0.25	0.2735
Prevalent TB (ref: No prevalent TB)	-0.57	0.29	0.047
Log_10_ viral load (copies/ml)	-0.61	0.11	<0.001
Time on ART (years)	1.89	0.37	<0.001
Time ×Prevalent TB (ref: no prevalent TB)	1.26	0.16	<0.001
Time × log_10_ viral load	0.19	0.07	0.007

^*a*^adjusted estimates; S.E.: standard error

**Table 3:** Event process estimates from the joint model and time-varying Cox model estimates

	*β* estimate	aHR (95% CI)	S.E.	*p*-value
**Time-varying proportional hazards model**				
Longitudinal $\sqrt {CD4}$ count (per unit decrease)	0.16	1.17 (1.12-1.23)	0.03	<0.001
**Event Process**				
Age (years)	0.01	1.01 (0.99-1.03)	0.01	0.148
Log_10_ viral load (copies/ml)	0.37	1.45 (1.21-1.69)	0.12	0.002
Men (ref: women)	0.05	1.05 (0.70-1.40)	0.18	0.775
Urban site (ref: rural)	-0.08	0.92 (0.49-1.35)	0.22	0.758
Prevalent TB (ref: no prevalent TB)	-0.21	0.81 (0.34-1.28)	0.24	0.377
**Association** (per unit decease)	0.30	1.34 (1.27-1.42)	0.03	<0.001

aHR: adjusted hazard ratios; S.E.: standard error

The joint model for longitudinal and time-to-event data has several advantages especially in clinical trials. In a survival analysis setting, where the covariate of interest is time-dependent, either the entire history of the covariate for every subject, or, minimally, measurements of the covariate at each time of disease occurrence for all subjects in the corresponding risk set, are necessary. This extensive measurement of covariate is rarely, if ever, executed and the values obtained are typically subject to measurement error. Thus by modelling the covariates over time, we can enhance the survival analysis since we can interpolate covariate values between the observed measurements to the specific times of disease occurrence, with the use of the entire covariate history of the subjects. Furthermore, according to [32], after accounting for measurement error, the standard error of the relative risk estimate will reflect correctly the uncertainty in the measurements of the covariate. Conversely, utilizing the survival data in the longitudinal model will yield improved longitudinal parameter estimates by allowing adjustment for informative right censoring of the repeated measurements by the disease process. Furthermore, the joint model allows for unequally spaced measurements, or missing covariate data and censoring of survival times. The fact that the joint model has the distinct advantage of simultaneously modelling two response variables (for example in this study, CD4 count and time-to-death) allows the researcher some degree of flexibility.

We found that after ART initiation the CD4 count increases and is influenced by measured covariates such as age, gender TB status and baseline viral load. Furthermore, gender and baseline viral load were found to be significant predictors of all-cause mortality. The joint model found a strong association between CD4 count measurement process and mortality which means that the full CD4 count history is a predictor of mortality. These results are in consonant with previous research [11, 33, 34].

## Conclusion

Joint modelling enabled the assessment of the effect of longitudinal CD4 count on mortality while correcting for shared random effects between longitudinal and time-to-event models. In biomedical research where measurements of various outcomes are taken over a time period in an attempt to understand patients’ health or the risk of an event occurring, the joint modelling approach will be the most useful tool to consider in an effort to link the longitudinal measurement process and time-to-event outcomes. In the era of universal test and treat, the evaluation of CD4 count is still crucial for guiding the initiation and discontinuation of opportunistic infections prophylaxis and assessment of late presenting patients. The CD4 count can also be used when immunological failure is suspected as we have shown that it is associated with mortality.

The joint modelling approach is likelihood based and assumes that the data is missing at random. Further work will involve sensitivity analysis to determine the impact of departures from this assumption.

## Supplementary information

**Additional file 1** Supplementary tables.

## Data Availability

Data will be made available upon request but access will be controlled and each request will be considered on a case by case basis.

## Abbreviations

aHR: Adjusted hazard ratio
ART: Antiretroviral therapy
CAPRISA: Centre for the AIDS programme of research in South Africa
CAT: CAPRISA AIDS treatment
CI: Confidence intervals
EM: Expectation-maximization
HIV: Human immunodeficiency virus
HR: Hazard ratio
IQR: Interquartile range
MRR: Mortality rate ratio
S.E.: Standard error
TB: Tuberculosis
