# Supplementary material for: Joint modelling of longitudinal and time-to-event data: an illustration using CD4 count and mortality in a cohort of patients initiated on antiretroviral therapy
Source: BMC Infect Dis. 2020 Mar 30;20:256. doi: 10.1186/s12879-020-04962-3 (PMC7106785; doi:10.1186/s12879-020-04962-3)
Supplement: Supplementary file 1 — Additional file 1 Supplementary tables. [file 12879_2020_4962_MOESM1_ESM.pdf]

Additional Files

Table S1: Baseline predictors of CD4 count evolution using linear mixed effects multivariable model

Effect	β estimate[a]	S.E.	p-value
Intercept	17.82	0.82	< 0.001
Age (years)	-0.02	0.01	0.047
Men (ref: women)	-1.90	0.22	< 0.001
Urban site (ref: rural)	-0.33	0.25	0.189
No prevalent TB (ref: prevalent TB)	0.71	0.31	0.022
Log_{10} viral load	-0.65	0.12	< 0.001
Time on ART (years)	2.67	0.39	< 0.001
Time $\times$ no prevalent TB (ref: prevalent TB)	-1.27	0.17	< 0.001
Time $\times$ log_{10} viral load	0.24	0.07	0.005

[a]adjusted estimates; S.E.: standard error

Table S2: Baseline predictors of mortality using Cox proportional hazards model

Effect	HR (95% CI)	p-value	aHR (95% CI)	p-value
Age (years)	1.01 (0.99-1.03)	0.191	1.01 (0.99-1.03)	0.237
Rural site	1.0		1.0	
Urban site	0.97 (0.66-1.43)	0.882	1.02 (0.68-1.55)	0.919
Women	1.0		1.0	
Men	1.69 (1.21-2.35)	0.002	1.62 (1.16-2.26)	0.005
No prevalent TB	1.0		1.0	
Prevalent TB	1.13 (0.75-1.71)	0.573	1.31 (0.84-2.05)	0.238
Log_{10} viral load (copies/ml)	1.57 (1.26-1.97)	< 0.001	1.57 (1.26-1.96)	0.004

aHR: adjusted hazard ratios
